# Response to the letter to the editor by Dr. Tadashi Yoshida: “long noncoding RNAs: new players regulating vascular calcification?”

**DOI:** 10.1080/0886022X.2020.1808480

**Published:** 2020-08-27

**Authors:** Weikang Guo, Wenhu Liu

**Affiliations:** Department of Nephrology, Beijing Friendship Hospital, Faculty of Kidney Diseases, Capital Medical University, Beijing, China

In reply to Professor Yoshida, we would like to thank Professor Yoshida for his interest in our article and the constructive comments [1].

Vascular calcification is prevalent in chronic kidney disease (CKD), which promotes death caused by cardiovascular events. High level of phosphorus (HP) can contribute to the induction of osteogenic differentiation of vascular smooth muscle cells (VSMCs) in CKD. It is likely that lncRNAs may play an important role in this process. In our study [2], we found 728 lncRNAs differentially expressed in HP group compared to the control group. LncRNA-mRNA co-expression network analysis revealed that 8 lncRNAs were the most highly connected lncRNAs. This study provided a candidate list of lncRNAs potentially involved in osteogenic differentiation, to some extent, providing a new insight into the research in the quest to comprehend molecular mechanisms involved in vascular calcification induced by HP in CKD.

We greatly agree and appreciate Professor Yoshida's comments and suggestions. Indeed, osteogenic transdifferentiation of VSMCs happens in an early time under HP stimulation. It would be worthy to detect mRNA or lncRNA changes in VSMCs in response to HP stimulation within a short phase. However, previous studies show that osteogenic transdifferentiation and calcification of VSMCs could be further aggravated after treating VSMCs with HP for 10 days and even 14 days [3,4], suggesting a persistent transdifferentiation process under HP pressure. Stimulation with HP attenuated the expression of lncRNA-GAS5 in human VSMCs in a time-dependent manner (day 0, 3, 7 and 14); lncRNA-GAS5 could inhibit HP-induced osteoblast differentiation and calcification *in vitro* [4]. Therefore, we think detecting the mRNA and lncRNA changes in VSMCs with HP incubation for 7 days can also provide crucial hints for further understanding of the potential mechanisms of HP-induced osteogenic transdifferentiation and calcification.

In our paper, the identified mRNAs and lncRNAs were differentially expressed in HP treated VSMCs compared to the control, which could be considered as HP stimulation related molecules involved in osteoblast differentiation and calcification. Actually, the specific roles and underlying mechanisms need to be further explored as Professor Yoshida’s kind suggestions. Gain-of-function or loss-of-function experiments to explore the identified mRNAs or lncRNAs of interest will be carried out in future studies. In the lncRNA-transcription factor mRNA network analysis, we also noticed that NF-κB was in the network. Professor Yoshida reported that NF-κB pathway acted an important role in CKD vascular calcification [5]. Besides, other researches also stress the role of NF-κB in CKD vascular calcification [6]. So it will be with great interest to investigate the roles of lncRNAs involved in the regulation of NF-κB in the vascular calcification process of CKD in future study.

Because *in vitro* experiments often do not reflect the real world as Professor Yoshida points out, we need to conduct *in vivo* experiments. We will further determine whether the identified mRNAs and lncRNAs fucntion in animal models of vascular calcification. Besides, Professor Yoshida mentioned that VSMCs have diverse embryological origins and different VSMCs possess different biological functions. In our paper, human aortic smooth muscle cells (HASMC) line was employed, which is the most commonly used cell line in the study of vascular calcification in CKD. It is will interesting to determine whether other SMCs derived from multiple embryological origins share common molecular mechanisms for vascular calcification.
